# Minimal Clinically Important Difference for Worsening of the University of California San Diego Shortness of Breath Questionnaire in Patients With Idiopathic Pulmonary Fibrosis With Mild or Moderate Impairment in Lung Function

**DOI:** 10.1016/j.chpulm.2025.100145

**Published:** 2025-02-07

**Authors:** Kerri I. Aronson, Ganesh Raghu, Sachin Gupta, Jinnie Ko, Jacob Devine, Jeffrey Swigris

**Affiliations:** a Division of Pulmonary and Critical Care Medicine, Weill Cornell Medicine, New York, NY; b Center for Interstitial Lung Diseases, University of Washington, Seattle, WA; c Genentech, Inc., South San Francisco, CA; d Center for Interstitial Lung Disease, National Jewish Health, Denver, CO

**Keywords:** dyspnea, IPF, minimal clinically important difference, University of California San Diego Shortness of Breath Questionnaire

## Abstract

**Background:**

The University of California San Diego Shortness of Breath Questionnaire (UCSD-SOBQ) is used commonly in clinical trials to evaluate dyspnea in patients with idiopathic pulmonary fibrosis (IPF). Previously, the minimal clinically important difference (MCID) for UCSD-SOBQ was estimated at 8 points for patients with IPF with severe impairment as determined by lung function tests.

**Research Question:**

What is the MCID threshold for UCSD-SOBQ worsening in patients with IPF and less physiologic impairment?

**Study Design and Methods:**

Pooled data from patients with baseline forced vital capacity (FVC) of ≥ 50% predicted enrolled in the 2 randomized phase 3 CAPACITY trials of pirfenidone (ClinicalTrials.gov Identifiers: NCT00287716 and NCT00287729) were analyzed. Over the 72-week treatment period, the MCIDs for the UCSD-SOBQ were estimated using anchor-based methods by comparing changes in UCSD-SOBQ scores with values for stability/improvement vs worsening in 4 candidate anchors: FVC, diffusing capacity of the lungs for carbon monoxide (Dlco), 6-minute walk distance (6MWD), and St George’s Respiratory Questionnaire activity domain (SGRQ-A). Anchors that correlated with UCSD-SOBQ scores for at least 1 measurement (correlation coefficient ≥ 0.3) were included for MCID determination in a receiver operating characteristics approach.

**Results:**

Overall, UCSD-SOBQ score changes were similar within stability/improvement and worsening categories for FVC, Dlco, and 6MWD anchors. For the SGRQ-A anchor, for stability/improvement, UCSD-SOBQ changes were lower than for the other anchors; for worsening, changes were greater. Based on suitable correlation coefficients, FVC, 6MWD, and SGRQ-A were the anchors used to triangulate the MCID. Optimal anchor cut points (MCID) for worsening were 6 for FVC, 4.3 for 6MWD, and 5.8 for SGRQ-A, which resulted in a *z*-transformed weighted average for UCSD-SOBQ worsening of 5.97.

**Interpretation:**

Our results show that in patients with IPF and mild or moderate physiologic impairment, the UCSD-SOBQ has a MCID for worsening of 4 to 6 with a point estimate of 6.

**Trial Registry:**

ClinicalTrials.gov; No.: NCT00287716 and NCT00287729; URL: www.clinicaltrials.gov

**Take-Home Points**

**Study Question:** What is the minimal clinically important difference (MCID) for worsening of the University of California San Diego Shortness of Breath Questionnaire (UCSD-SOBQ) score in patients with idiopathic pulmonary fibrosis with mild or moderate impairment in lung function from the 2 randomized phase 3 CAPACITY trials of pirfenidone (ClinicalTrials.gov Identifiers: NCT00287716 and NCT00287729)?

**Results:** Using an anchor-based receiver operating characteristics approach, the MCID estimate for UCSD-SOBQ score worsening was 6 points.

**Interpretation**: The estimated MCID for worsening of the UCSD-SOBQ score in this study can be used in future clinical trials in which UCSD-SOBQ is used as an end point, enabling the detection of a change in breathlessness that is meaningful to patients.

Dyspnea is a common and debilitating symptom of idiopathic pulmonary fibrosis (IPF) that has a substantial negative impact on patients’ quality of life and prognosis.^1^^,^^2^ Dyspnea scales provide tools for measuring this cardinal symptom.^3^, ^4^, ^5^, ^6^ The University of California San Diego Shortness of Breath Questionnaire (UCSD-SOBQ) is a 24-item patient-reported outcome (PRO) measure that has properties that render it acceptable for use as a measure of dyspnea in patients with IPF.^7^^,^^8^ It has been used as such in numerous clinical trials in patients with fibrotic interstitial lung disease (ILD).^9^, ^10^, ^11^, ^12^ The UCSD-SOBQ measures the severity of dyspnea during various activities (21 items scored from 0 to 5) and includes 3 additional questions on limitations associated with shortness of breath, fear of self-harm by overexerting, and fear of shortness of breath (3 items scored from 0 to 5). The scores are summed to give a total score ranging from 0 to 120, and a higher score is associated with greater severity of shortness of breath.^7^^,^^8^

Establishing a minimal clinically important difference (MCID; also known as the *meaningful score difference*^13^) for any PRO is important for interpreting change scores, including in response to treatment.^14^ The MCID for UCSD-SOBQ in IPF has been estimated to be 8 (range, 5-11) using baseline-to-24-week data from 180 patients with severe physiologic impairment (diffusing capacity of the lungs for carbon monoxide [Dlco] < 35% predicted) who were enrolled in the Sildenafil Trial of Exercise Performance in Idiopathic Pulmonary Fibrosis (STEP-IPF; ClinicalTrials.gov Identifier: NCT00517933) study.^8^^,^^15^ Because this MCID was estimated in a cohort of patients with advanced IPF (as determined by lung function), it is unclear whether this threshold is applicable in patients with more preserved lung function. A more recent MCID based on a population of patients with IPF with mild to moderate impairment who took part in the PANTHER-IPF trial (Prednisone, Azathioprine, and N-Acetylcysteine: A Study That Evaluates Response in Idiopathic Pulmonary Fibrosis) has been reported as 11.38 (95% CI, 7.83-14.93) over 60 weeks.^16^ However, a separate analysis of patients from a Canadian pulmonary registry produced an estimated MCID of 4.7 (range, 1.0-8.5) for the subgroup of 539 patients with IPF,^17^ and an observational, prospective multicenter study of 238 patients with IPF of mixed severity in the United Kingdom reported the UCSD-SOBQ MCID to be 9.6 (range, 4.1-14.2).^18^ The objective of the current analysis was to re-establish the MCID for worsening of the UCSD-SOBQ score using data from the large number of patients with IPF who were enrolled in the phase 3 CAPACITY trials and had mild or moderate physiologic impairment.

## Study Design and Methods

### Patients and Study Design

This analysis included pooled data from the phase 3 CAPACITY studies (studies 004 [ClinicalTrials.gov Identifier: NCT00287716] and 006 [ClinicalTrials.gov Identifier: NCT00287729]), 2 global, randomized, placebo-controlled, double-masked trials of pirfenidone in patients with IPF. Full details of the study design and eligibility criteria for CAPACITY have been published previously.^10^ Briefly, the trials enrolled patients 40 to 80 years of age with a confirmed diagnosis of IPF by high-resolution CT imaging with or without surgical lung biopsy, forced vital capacity (FVC) of ≥ 50% predicted, Dlco of ≥ 35% predicted, either FVC or Dlco ≤ 90% predicted, and 6-minute walk distance (6MWD) of ≥ 150 m. Exclusion criteria included the presence of obstructive airway disease or connective tissue disease, any alternative explanation for ILD, and being on a waiting list for a lung transplant.^10^ In study 004, patients were randomized 2:1:2 to pirfenidone 2,403 mg/d, pirfenidone 1,197 mg/d, or placebo, and in study 006, patients were randomized 1:1 to pirfenidone 2,403 mg/d or placebo. Using the UCSD-SOBQ, change in dyspnea from baseline was assessed every 12 weeks (until week 72), with mean change from baseline to week 72 as a secondary end point in both studies. The study protocols were approved by the institutional review board or ethics committee at each participating center, and all patients provided written, informed consent before participation. In this exploratory analysis, the MCID for UCSD-SOBQ was evaluated using pooled data from all patients with baseline FVC of ≥ 50% predicted enrolled in the CAPACITY trials.

### Determining Anchors for MCID Estimation

First, the Spearman correlation was used to examine the strength and direction of the relationship between baseline UCSD-SOBQ scores and baseline anchor values for each of 4 candidate anchors: FVC, Dlco, 6MWD, and St George’s Respiratory Questionnaire activity domain (SGRQ-A). Correlations between UCSD-SOBQ change scores and candidate anchor change scores also were assessed. Although we performed analyses for all anchors, anchors with at least 1 (baseline or change) correlation coefficient of ≥ 0.3 were included for MCID determination.

Based on published literature, we considered the following MCID values for candidate anchors: (1) for FVC, a < 5% decline in the raw value was categorized as stability/improvement, and a ≥ 5% decline was categorized as worsening^19^; (2) for Dlco, a < 10% decline in the raw value was categorized as stability/improvement, and a ≥ 10% decline was categorized as worsening^20^; (3) for 6MWD, a decline of < 24 m was categorized as stability/improvement, and a ≥ 24-m decline was categorized as worsening^21^; and (4) for SGRQ-A, a < 5-point increase was categorized as stability/improvement, and a ≥ 5-point increase was categorized as worsening.^22^^,^^23^

For each anchor, a receiver operating characteristic approach was used to estimate the UCSD-SOBQ cut point that best discriminated between stability/improvement and worsening using the Youden index. MCID estimates were triangulated using weighting based on the correlation between dichotomized anchor and UCSD-SOBQ change values.^24^ Finally, for each UCSD-SOBQ anchor pair, cumulative distribution function curves were generated to provide a visual representation of changes from baseline in UCSD-SOBQ scores for dichotomized anchor change subgroups (stability/improvement vs worsening at week 72). Distribution-based estimates for the MCID of UCSD-SOBQ worsening (e-Appendix 1) were generated in exploratory analyses; however, as suggested by the US Food and Drug Administration,^25^ the final estimation of the UCSD-SOBQ MCID was based only on results from the anchor-based analyses.^25^^,^^26^

## Results

### Patients

In total, 779 patients were included in the pooled CAPACITY population^10^; however, 1 patient was excluded from the current analysis because they did not meet the inclusion criterion of baseline FVC of ≥ 50% predicted. Key baseline characteristics of the 778 patients who were included are summarized in Table 1. Median age was 68.0 years, and most patients were male (71.6%) and White (97.4%). The mean (SD) FVC and Dlco were 74.9% predicted (14.4% predicted) and 46.9% predicted (9.5% predicted), respectively. The mean (SD) 6MWD was 401.6 (92.6) m, and the mean (SD) UCSD-SOBQ score was 33.5 (21.1).

**Table 1:** Baseline Characteristics in the CAPACITY Studies

Characteristic	CAPACITY (N = 778)^a^
Baseline characteristics	
Age, y	68.0 (40.0-81.0)
Male sex	557 (71.6%)
White	758 (97.4%)
Current or former smoking history	522 (67.1%)
Time since IPF diagnosis, y	1.25 (1.1) [n = 777]
FVC	
L	2.9 (0.7)
% predicted	74.9 (14.4)
Dlco	
mL/min/mm Hg	13.4 (3.5) [n = 710]
% predicted	46.9 (9.5) [n = 776]
6MWD, m	401.6 (92.6) [n = 764]
Oxygen use	165 (21.2%)
UCSD-SOBQ score	33.5 (21.1) [n = 763]
SGRQ-A score	36.9 (17.0) [n = 748]
Treatment during CAPACITY	
Pirfenidone	432 (55.5%)

Data are presented as No. (%), median (range), or mean (SD) unless otherwise indicated. 6MWD = 6-min walk distance; Dlco = diffusing capacity of the lungs for carbon monoxide; FVC = forced vital capacity; IPF = idiopathic pulmonary fibrosis; SGRQ-A = St George’s Respiratory Questionnaire activity domain; UCSD-SOBQ = University of California, San Diego Shortness of Breath Questionnaire.

a In total, 779 patients were included in the pooled CAPACITY population; however, 1 patient was excluded from this analysis because they did not meet the inclusion criterion of baseline FVC ≥ 50% predicted.

### Estimating MCID for UCSD-SOBQ

Baseline UCSD-SOBQ scores were correlated weakly with FVC % predicted (Spearman correlation coefficient, –0.22) and Dlco % predicted (Spearman correlation coefficient, –0.25), moderately correlated with 6MWD (Spearman correlation coefficient, –0.33), and strongly correlated with SGRQ-A (Spearman correlation coefficient, 0.81) (Table 2). In Table 3, changes from baseline to week 72 in UCSD-SOBQ scores are represented for dichotomized anchor subgroups. The magnitude of changes in UCSD-SOBQ scores generally was consistent across patients who showed stability/improvement in FVC, Dlco, and 6MWD and across patients with worsening of FVC, Dlco, and 6MWD. Compared with corresponding groups for FVC, Dlco, or 6MWD, patients with stability/improvement of SGRQ-A scores showed lower UCSD-SOBQ score changes, and patients with worsening of SGRQ-A scores showed higher UCSD-SOBQ score changes. Strength of correlation between anchor change and change from baseline to week 72 in UCSD-SOBQ was weak for 6MWD (Spearman correlation coefficient, –0.28), Dlco (Spearman correlation coefficient, –0.29), and FVC (Spearman correlation coefficient, –0.31) and was moderate for SGRQ-A (Spearman correlation coefficient, 0.53) (Table 3).

**Table 2:** Mean UCSD-SOBQ Scores per Anchor Quartile and Correlations Between Baseline UCSD-SOBQ and Anchor Scores

Anchor	No.	UCSD-SOBQ Score	Spearman Correlation Coefficient
FVC % predicted			–0.2225
< 64.1	194	40.1 (21.2)	
64.1 to < 73.4	195	34.9 (19.9)	
73.4 to < 83.6	194	30.0 (20.0)	
≥ 83.6	195	28.9 (21.5)	
Dlco % predicted			–0.2479
< 39.4	194	39.4 (21.4)	
39.4 to < 45.7	194	35.7 (20.3)	
45.7 to < 52.4	194	33.1 (21.5)	
≥ 52.4	194	25.9 (18.7)	
6MWD, m			–0.3347
< 345.0	190	43.1 (23.1)	
345.0 to < 400.0	188	36.1 (19.9)	
400.0 to < 460.0	194	30.3 (19.2)	
≥ 460.0	192	24.8 (17.9)	
SGRQ-A score			0.8054
< 36.7	190	13.0 (9.8)	
36.7 to < 53.6	169	25.9 (12.6)	
53.6 to < 66.5	211	37.6 (15.1)	
≥ 66.5	191	55.9 (17.1)	

Data are presented as mean (SD) unless otherwise indicated. 6MWD = 6-min walk distance; Dlco = diffusing capacity of the lungs for carbon monoxide; FVC = forced vital capacity; SGRQ-A = St George’s Respiratory Questionnaire activity domain; UCSD-SOBQ = University of California San Diego Shortness of Breath Questionnaire.

**Table 3:** Dichotomized Change From Baseline UCSD-SOBQ Scores and Anchor Values at Week 72, and Spearman Correlation Coefficient Between Change From Baseline to Week 72 in UCSD-SOBQ Scores and Change in Anchor Values

Change in Anchor	Change From Baseline to Week 72 in Anchor Values	Change in UCSD-SOBQ Score	Spearman Correlation Coefficient
FVC			–0.3127
Stability/improvement	308 (45.5%)	2.8 (14.6)	
Worsening	269 (54.5%)	11.5 (18.4)	
Dlco			–0.2911
Stability/improvement	267 (40.0%)	2.4 (15.8)	
Worsening	401 (60.0%)	10.8 (17.5)	
6MWD			–0.2843
Stability/improvement	336 (51.7%)	3.0 (14.9)	
Worsening	314 (48.3%)	11.6 (18.8)	
SGRQ-A			0.5290
Stability/improvement	316 (49.0%)	0.5 (15.3)	
Worsening	329 (51.0%)	13.9 (16.6)	

Data are presented as No. (%) or mean (SD) unless otherwise indicated. 6MWD = 6-min walk distance; Dlco = diffusing capacity of the lungs for carbon monoxide; FVC = forced vital capacity; SGRQ-A = St George’s Respiratory Questionnaire activity domain; UCSD-SOBQ = University of California San Diego Shortness of Breath Questionnaire.

**Figure 1:**
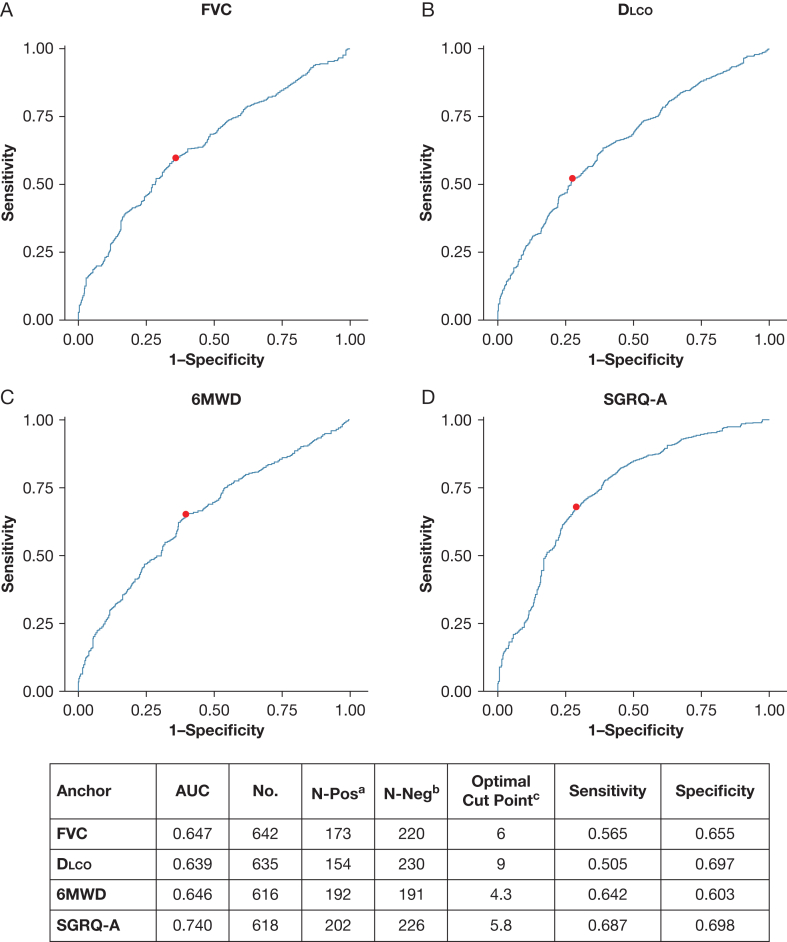
Receiver operating characteristic curves for test characteristics and optimal cut points of UCSD-SOBQ scores for identifying worsening, stability, or improvement of dyspnea symptoms according to FVC (A), Dlco (B), 6MWD (C), and SGRQ-A (D) anchors. ^a^The number of patients who have UCSD-SOBQ scores showing stability/improvement as well as anchor results showing stability/improvement. ^b^The number of patients who have UCSD-SOBQ results showing worsening as well as anchor results showing worsening. ^c^Data taken from the Youden index. 6MWD = 6-min walk distance; AUC = area under the receiver operating characteristic curve; Dlco = diffusing capacity of the lungs for carbon monoxide; FVC = forced vital capacity; SGRQ-A = St George’s Respiratory Questionnaire activity domain; UCSD-SOBQ = University of California San Diego Shortness of Breath Questionnaire.

On receiver operating characteristic analysis, the Dlco anchor showed the highest Youden index (Fig 1). However, because Spearman correlation coefficient values between Dlco and both baseline and change scores for UCSD-SOBQ were < 0.3, Dlco was not included as an anchor in the MCID estimation. FVC, 6MWD, and SGRQ-A, which all had correlation coefficients of > 0.3 for at least 1 measure, were used as the anchors to triangulate the MCID for UCSD-SOBQ. The optimal cut points (MCIDs) for UCSD-SOBQ score worsening from baseline at 72 weeks were 6 for FVC, 4.3 for 6MWD, and 5.8 for SGRQ-A, which yielded a *z*-transformed correlation-weighted point estimate of 5.97 (95% CI, 2.58-9.37), that is, 6 points.

**Figure 2:**
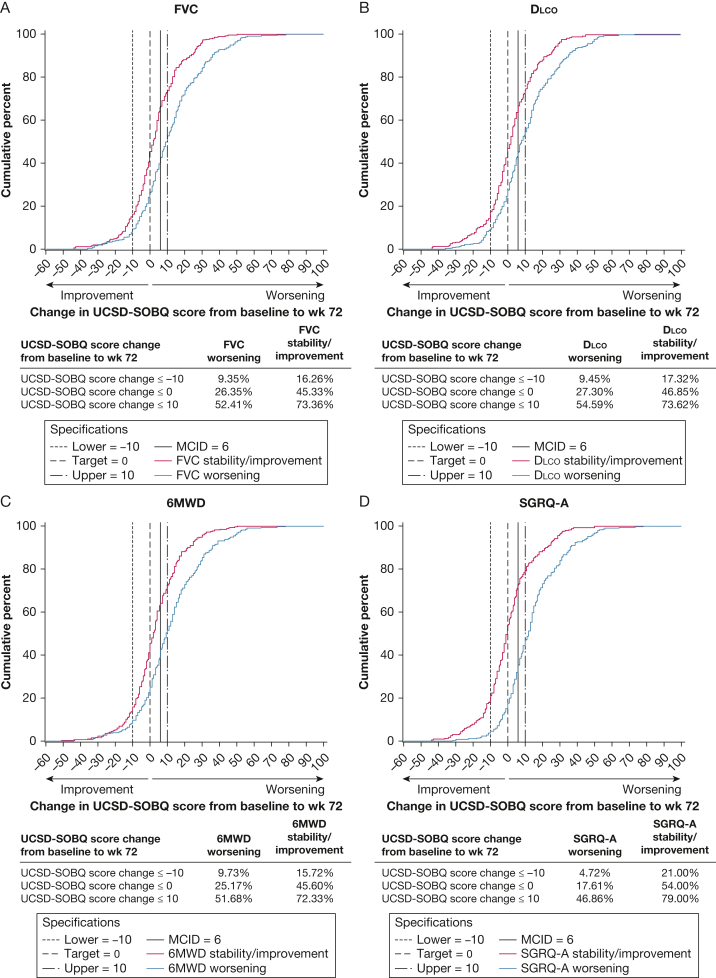
Graphs showing cumulative distribution functions for change in UCSD-SOBQ scores at week 72: FVC (A), Dlco (B), 6MWD (C), and SGRQ-A (D). 6MWD = 6-min walk distance; Dlco = diffusing capacity of the lungs for carbon monoxide; FVC = forced vital capacity; MCID = minimal clinically important difference; SGRQ-A = St George’s Respiratory Questionnaire activity domain; UCSD-SOBQ = University of California San Diego Shortness of Breath Questionnaire.

Cumulative distribution function curves for UCSD-SOBQ score changes anchored on dichotomized FVC, Dlco, 6MWD, and SGRQ-A values at week 72 are shown in Figure 2. The figures for each anchor show separation of the two anchor subgroup curves (worsened vs stable/improved) across the range of SOBQ change scores.

Analyses of MCID using distribution-based methods are presented in e-Appendix 1.

## Discussion

In the current analysis, we explored an anchor-based receiver operating characteristic approach to generate the MCID threshold for worsening of UCSD-SOBQ scores. Estimated MCIDs were 6 for FVC, 4.3 for 6MWD, and 5.8 for SGRQ-A, leading to a *z*-transformed correlation-weighted point estimate of 5.97 (6 points). This value is lower than the estimate generated for patients with IPF with more severe impairment in lung function tests.^8^ Our results suggest that smaller changes than previously estimated may be clinically meaningful and may reflect patients’ perception of deterioration in dyspnea. Given that IPF is a progressive disease without any current management options that have demonstrated disease modification or improvement in dyspnea over time, we focused on the MCID for worsening, rather than improvement in, dyspnea. However, future studies may wish to assess the MCID for improvement in UCSD-SOBQ as management options evolve (for example, with opioid-based therapies or pulmonary rehabilitation).

Psychometric properties of UCSD-SOBQ, including a linear regression-derived estimate of the MCID for worsening of 8 points (range, 5-11) at 24 weeks, originally were explored in the STEP-IPF cohort, which included 180 patients with more advanced IPF.^8^ More recently, in a heterogeneous registry cohort of patients with various forms of ILD, Chen and colleagues^17^ also used linear regression to derive an MCID threshold for worsening of 4.7 (range, 1.0-8.5) in the subgroup of patients with IPF, who had similar baseline characteristics as those in the CAPACITY population. A larger MCID of 11.38 (95% CI, 7.83-14.93) was estimated based on the PANTHER-IPF trial in patients with IPF with mild to moderate impairment using an anchor-only approach, albeit 1 with a single rating of change measure.^16^ However, this single anchor has a recall period of 1 year, which makes it prone to recall bias, and it may not be well suited to anchoring other durations of changes.^16^ A prospective study by Kim and colleagues^18^ of data from 238 patients with varying degrees of IPF severity reported the MCID to be 9.55 (95% CI, 2.97-16.14) using anchor-based methods, with various PRO measures, 6MWD, and FVC % predicted as anchors. In the current analysis of the CAPACITY cohort—the largest to date—we build on findings from previous studies by estimating the MCID threshold for worsening over a longer period in patients with mild or moderate impairment in lung function measurements. Although the anchors and cut points for FVC, Dlco, and 6MWD in the current analysis were slightly different from the those of the STEP-IPF and Chen and colleagues analyses, our point estimate for worsening (6 points) falls within the ranges for estimates in both studies.

Looking more closely at the individual anchors, it was noted that the correlations between UCSD-SOBQ score and the selected anchors were stronger for the PRO, that is, SGRQ-A (Spearman correlation coefficient, 0.81), than for 6MWD (Spearman correlation coefficient, –0.33) and the physiologic measures of FVC % predicted (Spearman correlation coefficient, –0.22) and Dlco % predicted (Spearman correlation coefficient, –0.25). These results align with previous analyses in which UCSD-SOBQ also correlated more strongly with PROs than with physiologic measures among populations of patients with IPF or ILD.^8^^,^^17^^,^^18^ For example, in patients with fibrotic ILD from the Canadian Registry for Pulmonary Fibrosis, Spearman correlation coefficients of UCSD-SOBQ score with other PROs were 0.83 (SGRQ), –0.74 (EuroQol five-dimension five-level [EQ-5D-5L]), and –0.59 (EuroQol visual analogue scale [EQ-VAS]) vs –0.48 for 6MWD, –0.41 for Dlco, and –0.40 for FVC.^17^ Similarly, UCSD-SOBQ score correlations using data from the STEP-IPF trial were strong for PROs (Spearman correlation coefficients: 36-item short-form health survey [SF36] physical functioning domain, –0.72; SGRQ-A domain, 0.80), moderate for 6MWD (Spearman correlation coefficient, –0.39), and weak for FVC % predicted (Spearman correlation coefficient, –0.22) and Dlco % predicted (Spearman correlation coefficient, –0.20).^8^ In addition, Kim and colleagues^18^ found strong correlations with PROs (Spearman correlation coefficients: SGRQ, 0.83; EuroQol five-dimension [EQ-5D], –0.78; Medical Research Council dyspnea score, 0.74; Hospital Anxiety and Depression Scale, 0.6; King’s Brief Interstitial Lung Disease questionnaire, –0.74; and IPF-specific SGRQ, 0.822), but moderate correlation with 6MWD (Spearman correlation coefficient, –0.34) and weak correlation with FVC % predicted (Spearman correlation coefficient, –0.28). A similar pattern was observed for 12-month real-world data correlations of IPF-specific SGRQ and King’s Brief Interstitial Lung Disease questionnaire with PRO-based anchors of global rating of change scales and change in UCSD-SOBQ and SGRQ scores vs those for FVC % predicted, Dlco % predicted, and 6MWD in patients with IPF.^27^ Taken together, these findings indicate a need for better physiologic anchors for estimating dyspnea-related MCIDs in patients with IPF; however, such analyses are limited by the measures used in registries and clinical trials. The wider inclusion of PROs, including fit-for-purpose anchors, in future studies would provide an opportunity for the re-evaluation of MCIDs with potentially more robust estimates in the future.

The main limitation of this analysis is that the data are from a randomized clinical trial; thus, considering that patients with IPF met precise inclusion criteria, results may not apply to real-world patient populations with IPF, especially those with comorbidities such as emphysema or forced expiratory volume in 1 second (FEV_1_) to FVC ratio of < 0.75 who may be excluded from clinical trials.^9^^,^^28^ However, this should be weighed against the potential advantage of reduced bias through systematic and consistent data collection in trials. The pooling of data from multiple trials also comes with inherent limitations, such as increasing the variability and heterogeneity of the population^29^; however, in this instance, because of the similar study designs, we believe that pooling is appropriate. Because of the retrospective and exploratory nature of this analysis, measures were not chosen as anchors a priori, and apart from SGRQ-A, were not tied to the patient perspective. The inclusion of more patient-centered anchors measuring similar constructs (for example, patient global impression of severity and change in dyspnea) would have alleviated the need for relying on physiologic anchors. Similarly, respiratory-related hospitalization could be considered for inclusion as an anchor in future studies, but was not included in the current analysis because of the low frequency of events in clinical trials of pirfenidone.^30^ Furthermore, most patients who participated in the CAPACITY trials were non-Hispanic, White, and male, and given the ethnic, cultural, and linguistic considerations for PROs,^31^ this lack of patient diversity may limit the generalizability of the results to the broader population. Although the 72-week time point used in the current analysis was appropriate when considering the long-term progression with IPF, future analyses looking at the MCID over time and at multiple shorter time points may offer additional value to patients. Evaluating relative change in UCSD-SOBQ, in addition to the absolute change measure in the current analysis, also would provide additional context to interpreting changes in dyspnea.

## Interpretation

We used data from a well-defined cohort of patients with IPF enrolled in the CAPACITY trials to estimate the MCID threshold for worsening of UCSD-SOBQ score at 6 points (range, 4-6). Although the results from these retrospective, exploratory analyses provide useful insights for the interpretation of the findings from clinical studies and can aid in the design of future studies incorporating the UCSD-SOBQ as an end point, the MCID estimate for the UCSD-SOBQ for patients with IPF and mild or moderate lung function impairment will need to be validated further in prospective studies.

## Funding/Support

This research was funded by 10.13039/100007013F. Hoffmann-La Roche, Ltd./10.13039/100004328Genentech, Inc. K. I. A. is supported by the 10.13039/100000050National Heart, Lung, and Blood Institute, National Institutes of Health [Grant K23 HL163394], the 10.13039/100002590American Lung Association, the 10.13039/100001844Scleroderma Foundation, and the Stony-Wold Herbert Foundation.

## Financial/Nonfinancial Disclosures

The authors have reported to *CHEST Pulmonary* the following: G. R. has received consulting fees from BMS, United Therapeutics, and Veracyte; has participated on a data safety monitoring board or advisory board for Avalyn (unpaid); has held unpaid leadership or fiduciary roles for the American Thoracic Society (IPF guideline chair) and the Pulmonary Fibrosis Foundation; has been an unpaid consultant for Bellerophon Therapeutics, F. Hoffmann-La Roche/Genentech, FibroGen, Gilead Sciences, Nitto BioPharma, and Novartis; not related to this study, has received as grants or contracts from National Heart, Lung, and Blood Institute/National Institutes of Health; and has been a scientific reviewer for investigator-initiated grant proposals for Boehringer Ingelheim. S. G., J. K., and J. D. are employees of Genentech, Inc. J. S. reports consultancy fees from AbbVie, BMS, Boehringer Ingelheim, Brainomix, Click Therapeutics, CSL Behring, Pliant, and Tvardi; stock ownership in Tvardi; participation in the medical advisory boards for patientMpower and PF Warriors. None declared (K. I. A.).

## Supplementary Data

e-Online Data
