# Supplementary material for: Minimal Clinically Important Difference for Worsening of the University of California San Diego Shortness of Breath Questionnaire in Patients With Idiopathic Pulmonary Fibrosis With Mild or Moderate Impairment in Lung Function
Source: CHEST Pulm. 2025 Feb 7;3(3):100145. doi: 10.1016/j.chpulm.2025.100145 (PMC13417585; doi:10.1016/j.chpulm.2025.100145)
Supplement: e-Online Data [file mmc1.pdf]

Minimal Clinically Important Difference for Worsening of the University of California San Diego Shortness of Breath Questionnaire in Patients With Idiopathic Pulmonary Fibrosis With Mild or Moderate Impairment in Lung Function

Kerri I. Aronson, MD, Ganesh Raghu, MD, Sachin Gupta, MD, Jinnie Ko, PhD, Jacob Devine, MA, Jeffrey Swigris, DO

e-Appendix 1

Determination of MCID for UCSD-SOBQ Worsening Using Distribution-Based Methods

To support/aid in interpreting the anchor-based estimates, minimal clinically important difference (MCID) thresholds were also derived via distribution-based methods: 1) standard error of measurement (with Cronbach's alpha as the reliability variable); and 2) consideration of the Cohen moderate effect size using baseline University of California San Diego Shortness of Breath Questionnaire (UCSD-SOBQ) data by dividing the standard deviation (SD) for the baseline domain score by either 2 or 5 for moderate and small effect size, respectively. For both anchor-based and distribution-based methods, analyses were performed using observed data only, and there was no imputation for missing values.

MCIDs calculated using distribution-based methods based on baseline UCSD-SOBQ data, which were evaluated as supportive data only, were 3.95 for Cronbach's alpha, and 10.560 (SD/2) and 4.224 (SD/5) for Cohen moderate effect size.
